# Exploring Acrylamide and 5-Hydroxymethylfurfural Formation in Glucose-Asparagine-Linoleic Acid System With a Kinetic Model Approach

**DOI:** 10.3389/fnut.2022.940202

**Published:** 2022-06-23

**Authors:** Yingjie Ma, You Long, Feng Li, Yan Zhang, Bei Gan, Qiang Yu, Jianhua Xie, Yi Chen

**Affiliations:** ^1^State Key Laboratory of Food Science and Technology, Nanchang University, Nanchang, China; ^2^Jiangxi Provincial Product Quality Supervision Testing College, Nanchang, China

**Keywords:** acrylamide, 5-hydroxymethylfurfural, kinetics, Maillard reaction, glucose-asparagine-linoleic acid

## Abstract

In the “glucose-asparagine-linoleic acid” ternary system, a kinetic model approach was used to explore formation and elimination law of target hazards, including acrylamide (AA) and 5-hydroxymethylfurfural (5-HMF), and their related precursors and intermediate products. The results showed that the elimination of glucose and asparagine and the formation of fructose (generated from glucose isomerization), 3-deoxyglucosone (3-DG), methylglyoxal (MGO), and glyoxal (GO), AA and 5-HMF followed first-order reaction kinetics with high fit coefficients (R^2^ > 0.9). In addition, the kinetic reaction rate constants increased as the increasing temperature, and all models followed the Arrhenius law. Results of statistical correlations analysis suggested that at lower temperature, the generic amino acid route and the specific amino acid route may paly crucial roles for the formation of AA and 5-HMF, while at high temperature a linoleic acid pathway may be predominantly involved.

## Introduction

During the thermal processing of foods rich in sugar, amino acids and oil, parallel and consecutive reactions take place, including Maillard reaction, caramelization, and lipid oxidation. In Maillard reaction, aldoses (e.g., glucose) or ketoses (e.g., fructose) have been reported to condense with amine groups to form Schiff bases, which are very unstable and can be cyclized into N-substituted aldoglycamine or N-substituted ketosylamine. They were then rearranged to Amadori products (1-amino-1-deoxy-ketose) or Heyns products (2-amino-2-deoxy-aldose) (1). Besides, it has been reported that fructofuranosyl cations react with amino compounds to form fructofuranosyl amines and rearrange to form Heyns products (2). Meanwhile, the degradation of Amadori or Heyns products is an important step in the reaction, resulting in α-dicarbonyl compounds formation (3, 4). For the caramelization reaction, it is the process by which aldoses or ketoses degrade at high temperature in the absence of amino compounds to form brown substances, and α-dicarbonyl compounds can be formed from sugar molecules during dehydration and degradation. In addition, some studies demonstrated that lipids can also produce many low molecular weight carbonyl compounds including α-dicarbonyl compounds via oxidative degradation or heat treatment (5).

α-Dicarbonyl compounds are important reactive intermediates in carbohydrate degradation. 3-deoxyglucosone (3-DG), methylglyoxal (MGO), and glyoxal (GO) as the main α-Dicarbonyl compounds can react with asparagine to form acrylamide (AA) via Strecker degradation (6). Additionally, the removal of two water molecules by 3-DG has been reported to lead to the formation of 5-hydroxymethylfurfural (5-HMF) (7, 8). The IARC has classed AA as a “probable human carcinogen” since it possesses genotoxic and carcinogenic characteristics, as well as neurotoxic properties at high doses (9). Studies have shown that 5-HMF has mutagenic effect on DNA, and cytotoxic effects on eyes, skin, upper respiratory tract and mucous membranes (10, 11).

More and more studies have been done to explore the involvement of Maillard and camalization reaction in the formation mechanism of AA or 5-HMF in the model system. De Vleeschouwer et al. (12, 13) reported the formation/elimination kinetics of acrylamide in asparagine-glucose systems by changing the initial reactant concentration and ratio and sugar types. Kocadagli et al. (14) explored the kinetics of the formation and degradation of α-dicarbonyl compounds in glucose/wheat flour system heated under low moisture conditions. These results have also been confirmed in real food systems. Berk et al. (15) explored the AA and 5-HMF formation mechanism during roasting of sesame seeds. Hamzalioglu et al. (16) reported that 5-HMF was a key intermediate during AA formation in the coffee system. However, these studies are focused on the Maillard reaction and caramelization, and the synergistic effect of lipid oxidation pathways has not been explored. In addition, kinetic modeling has received increasing attention in food research fields. It can reflect chemical changes in food, such as changes in color over time and temperature. If the rate and temperature dependence of the reaction is known, the occurrence of the reaction can in principle be predicted and controlled. Furthermore, it helps to understand the chemical nature and mechanism of the reaction (17).

Thus, in this study, the “glucose-asparagine-linoleic acid” ternary model system was applied to explore the simultaneous involvement of lipid oxidation and Maillard reaction in the formation of target hazards (AA and 5-HMF) through kinetic studies. It will give insights to evaluate the levels of α-dicarbonyl compounds including 3-DG, MGO, GO and hazards including AA and 5-HMF from food rich in carbohydrate, amino acids and unsaturated fatty acids. It also concerned about the relation of 5-HMF conversion into AA during thermal processing.

## Materials and Methods

### Chemicals

Standards for AA, 5-HMF, ^13^C_3_-AA, ^13^C_6_-HMF, 3-deoxyglucosone, quinoxaline, 2-methylquinoxaline, *o*-phenylenediamine (OPD),glucose and frucose were purchased from Sigma-Aldrich (St. Louis, MO, USA.). L-asparagine analytical standard was purchased from Beijing Solarbio Technology Co., Ltd (Beijing, China). HPLC-grade methanol and acetonitrile were obtained from Merck Company (Darmstadt, Germany). HPLC-grade formic acid was obtained from Shanghai Anpel Technology Co., Ltd (Shanghai, China). Distilled water was purchased from Watsons Water Co., Ltd. (Guangzhou, China). HPLC-grade n-heptane, D-glucose, asparagine and linoleic acid were obtained from Aladdin Biochemical Technology Co. Ltd. (Shanghai, China). *O*-phthalaldehyde (OPA) and 2-mercaptoethanol (2-ME) were purchased from Macklin Biochemical Technology Co., Ltd (Shanghai, China).

### Preparation of Model System

The model systems were prepared in 15 mL thick-walled pressure-resistant glass tubes (Beijing Synthware Glass Instrument Co. LTD, Beijing, China) with a sugar, asparagine, and oil mass ratio of about 15:13:4 to simulate the mass ratio used in biscuit manufacture (18), which contained 60 mg of glucose, 50 mg of asparagine, 16 mg of linoleic acid, and 2 mL of 0.1 M phosphate buffer (pH 7.4). The tubes were sealed with a polytetrafluoroethylene screwcap with a sealing ring and heated for 1, 3, 5, 7, 10, and 15 min in an oil bath (HH-SJ, Changzhou Jintan Youlian Instrument Research Institute, Changzhou, China) at 160, 180, and 200°C, respectively, then rapidly cooled in an ice bath.

### Analysis of Sugars

The analysis was conducted using an Agilent 1260 high performance liquid chromatography (HPLC) connected to a refractive index detector condition (RID). Separation of the analytes was performed on a CNW Athena NH_2_-RP column (4.6 × 250 mm, 5 μm, Shanghai Anpel Technology Co., Ltd, Shanghai, China) at 40 °C using an isocratic elution with a mobile phase consisting acetonitrile and distilled water (v/v = 70:30) at a flow rate of 1 mL/min and the injection volume was 20 μL.

### Analysis of Asparagine

Asparagine was analyzed by an Agilent 1290 HPLC system equipped with a diode array detector (DAD). OPA-2-ME reagent was prepared by a method described in a previous study (19). For precolumn derivatization, 20 μL of sample or 20 μL of asparagine standard was mixed with 30 μL of OPA-2-ME reagent and 150 μL of 0.1 M Na_2_B_4_O_7_ at room temperature for 3 min and passed through a 0.45 μm filter membrane waiting for analysis. The mobile phase consists of 0.01 M sodium phosphate buffer (pH 6.85) (A) and methanol (B) following a gradient elution procedure as previously reported by Yuan et al. (20). Samples were injected (20 μL) at 30 °C onto a Kromasil C_18_ column (250 × 4.6 mm, 5 μm; Akzo Nobel Pulp and Performance Chemicals, Bohus, Sweden) with the flow rate of 0.8 mL/min and the detection wavelength was 340 nm.

### Analysis of Linoleic Acid

The oil samples in the model system were extracted with petroleum ether and placed in a centrifuge tube. Then 2 mL of n-heptane and 0.1 mL of 2 M potassium hydroxide-methanol solution were added respectively. After mixing with a vortex mixer for 30 s, centrifuge at 4,800 rpm for 5 min, and take 1 mL of supernatant through a 0.22 μm filter for injection.

Analysis of linoleic acid was carried out using Agilent 8860 gas chromatography (GC) equipped with a flame ionization detector (FID) and a DB-FATWAX UI column (0.25 mm × 3 m, 0.25 μm; Agilent Technologies Inc., Santa Clara, CA, USA). The oven temperature program was as follows: 60°C held for 3 min, then increased to 170°C at 35 °C/min, held for 5 min, followed by heating up to 180°C at 5°C/min, held for 15 min, and finally increased at a rate of 5°C/min to 200°C. The helium flow rate was maintained constant at a flow rate of 1 mL/min and shunt ratio was 10:1.

### Analysis of AA and 5-HMF

The determination of AA and 5-HMF was performed according to the method described in a previous study (21). An Agilent ultra-high performance liquid chromatography system conjunction with triple-quadrupole mass spectrometer (LC-QqQ-MS/MS) coupled with electrospray ionization (ESI) source was used to conduct the analysis (Agilent Technologies Inc., Santa Clara, CA, USA). Separation of the analytes was performed on a Phenomenex Synergi Hydro-RP column (150 × 2.0 mm, 4 μm; Phenomenex, Torrance, CA, USA) with temperature of the column thermostat maintained at 30 °C. As mobile phase, methanol (A)/0.1% (V/V) formic acid in distilled water (B) were used at a flow rate of 0.3 mL min^−1^. The gradient elution was achieved under the following conditions: the program began at 95% B and was reduced to 80% B from 0 to 3 min; returned to 95% B over 5 min; and it was held at 95% B for 2 min. The injection volume was 1 μL. AA and 5-HMF were identified and quantified by means of multiple reaction monitoring (MRM) mode set to record the transitions m/z 72 → 55 and 72 → 27 for AA, m/z 75 → 58 and 75 → 29 for ^13^C_3_-AA, m/z 127 → 109 and 127 → 81 for 5-HMF and 133 → 115 and 133 → 86 for ^13^C_6_-HMF.

### Analysis of α-Dicarbonyl Compounds

#### Derivatization Procedure

200 μL of supernatant were combined with 400 μL of potassium phosphate buffer (pH 7.0) and 400 μL of 10 mg/mL OPD (in water), then filtered through 0.22 μm filters into an autosampler vial and stored at 25 °C for 3 h before detemination.

#### LC-QqQ-MS/MS Analysis

The α-dicarbonyl compounds were analyzed using an Agilent LC-QqQ-MS/MS with ESI source. 3-DG, MGO and GO quinoxaline derivatives were chromatographically separated on a Phenomenex Synergi Hydro-RP column (150 × 2.0 mm, 4 μm; Phenomenex, Torrance, CA, USA) at 30 °C. The mobile phase consisted of methanol/0.1% (V/V) formic acid in distilled water [60/40 (v/v)] at a flow rate was 0.25 mL/min with time of 10 min. The injection volume was 1 μL. Analytes were obtained using MRM mode with the ions monitored in the sample: m/z 235 → 199 and 235 → 171 for 3-DG, m/z 145 → 77 and 145 → 92 for MGO and m/z 131 → 77 and 131 → 51 for GO.

### Kinetic Modeling

The kinetic models were conducted using Origin 2019 (Microcal Software, Inc., Northampton, MA, USA)

The temperature dependence of the reaction is described by the activation energy E_a_ (kJ/mol) by establishing the Arrhenius equation:


$$\begin{array}{l} k\left(T\right)\;=\;A\;\times \;exp\;\left(\frac{E_{a}}{RT}\right), \end{array}$$

where k is the reaction rate constant, A is the pre-exponential factor, E_a_ is the activation energy (J mol^−1^), R is the universal gas constant (8.3145 kJ/mol K), and T is the temperature concerned.

### Statistical Analysis

All the experiments were performed at least triplicate and data was statistically analyzed using IBM SPSS statistical software (version 26.0, SPSS Inc., Chicago, IL, USA) and present as mean value ± SD (standard deviation) to determine the statistical significance. Analysis of variance (ANOVA) and Duncan's test were used to determine the differences between means, with a significance level of *P* < 0.05.

## Results and Discussion

### Kinetic Changes of Glucose, Frucose, Asparagine and Linoleic Acid Concentrations in Model System

**Figure 1:**
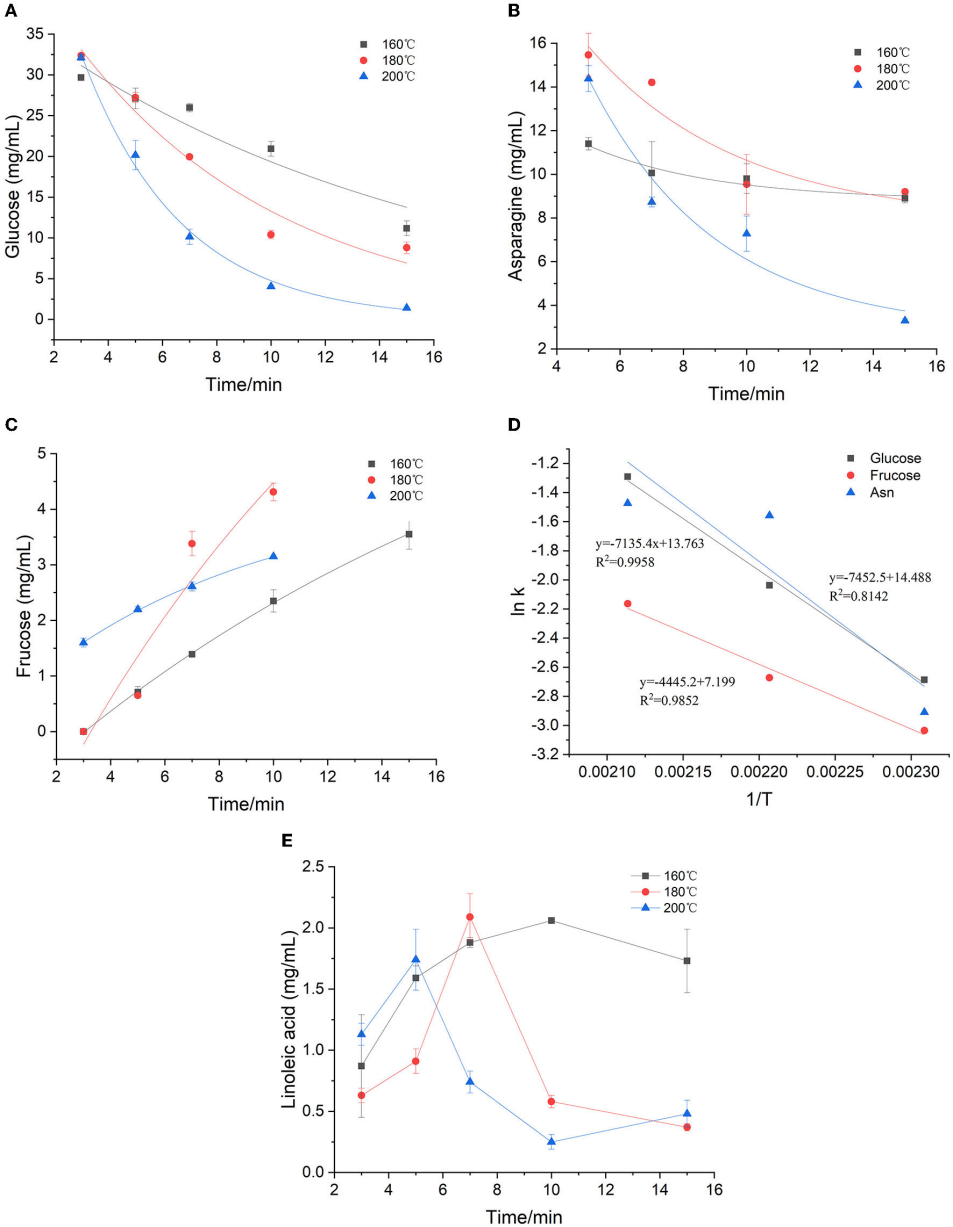
Kinetic changes of precursors in heated model system **(A)** glucose, **(B)** asparagine, **(C)** fructose, **(D)** Arrhenius plot and **(E)** Effect of time and temperature on linoleic acid concentration.

During the heating process, the concentration of glucose and asparagine, as the main precursors, decreased gradually with the increase of temperature and time, showing a trend of elimination kinetics (Figures 1A,B). Glucose decreased to the lowest concentration after heating for 15 min at 160, 180, and 200°C, respectively. Especially, the glucose was almost depleted when heated for 15 min at 200°C. The loss of glucose was not only a result of its reaction with asparagine, but also partly due to its participation in the isomerization reaction to form fructose in the heated sample (Figure 1C). By comparison, during the heating process, fructose was continuously formed. Furthermore, we found that fructose appeared to be the only detectable product of glucose isomerization under the applied reaction conditions, which was consistent with observations by other researchers (22, 23). At 160 and 180°C, the fructose concentration gradually increased with the increase of time and temperature, and reached the highest concentration at 15 min. While at 200°C, the concentration of fructose in the early stage of the reaction was more than three times higher than that of low temperature, but after 7 min, the reaction rate began to flatten out, which may be due to the further reaction of fructose with other compounds at higher temperature. It's been found that asparagine decreased slowly at 160°C, while decreased sharply at 180 and 200°C. It is reported that the concentration change of amino compounds was actually the result of at least three reactions: the reaction with sugar in the initial stage of the Maillard reaction; the regeneration of the amino compounds in the intermediate stage and the participation of the amino group in the browning reaction in the final stage (24). As shown in Figure 1B, a clear continuous loss of asparagine was observed when high temperature was applied. Meanwhile, it was found that the rate of asparagine loss was slower than that of glucose, which was consistent with the results of a glucose-asparagine system by Knol et al. (23). It can be explained by the regeneration of asparagine from the initial condensation product (such as the Amadori rearrangement product) and the possible formation of diglucosamine (22).

The first-order kinetic equation (Exp2PMod1) was used to fit the data of glucose in the model system. As for fructose and asparagine, their formation rates conformed to the first-order kinetic equation (Exponential). As shown in Figures 1A–C, the coincidence of the kinetic model curve with the actual data points showed that the model can accurately describe the glucose concentration at the three temperatures. Kinetic parameters were calculated by fitting the measured glucose values, as shown in Table 1. The fitting model of glucose obtained R^2^ > 0.897, indicating that the first-order kinetic model can be well described its concentration changes. Except that R^2^ at 180 °C was slightly lower (0.771 and 0.734) for fructose and asparagine, and R^2^ > 0.998 and 0.844 at 160 and 200°C, which could accurately describe the reaction process. As the baking temperature increased, the degradation rate constants of glucose and asparagine increased from 0.068 min^−1^ to 0.275 min^−1^ and from 0.054 to 0.229 min^−1^, indicating that the higher the temperature, the faster the reaction rate. In a previous study on a glucose/wheat flour system, the reaction rate constants for glucose at 160–200 °C ranged from 0.048 to 0.204 min^−1^ (14), in an order of magnitude with our results. The formation rate constants of fructose at three temperatures were 0.045, 0.069 and 0.115 min^−1^, respectively, which were slower than glucose and asparagine. The Arrhenius plot was plotted with the natural logarithm of the rate constant (ln k) vs. the inverse of the absolute temperature (1/T) (Figure 1D), which showed that ln k increased linearly as a function of 1/T. According to the slope of the linear equation, the calculated E_a_ of glucose and asparagine was 59.32 kJ/mol and 61.96 kJ/mol, while E_a_ of fructose was 36.96 kJ/mol, indicating that fructose formation was easier to be achieved compared to glucose and asparagine.

**Table 1:** Parameter of kinetics of glucose, fructose, and asparagine in heated model system.

	**Temperature (**°**C)**	**k**	**R^**2**^**	**E_**a**_ (kJ/mol)**
Glucose	160	0.068	0.897	59.32
	180	0.130	0.951	
	200	0.275	0.993	
Frucose	160	0.048	0.999	36.96
	180	0.069	0.771	
	200	0.115	0.998	
Asparagine	160	0.054	0.844	61.96
	180	0.211	0.734	
	200	0.229	0.875	

As shown in Figure 1E, the concentration of linoleic acid increased at the beginning and then decreased at three temperatures. Meanwhile, the higher the temperature, the earlier the drop point arrived, the faster the concentration decreased. At 160, 180, and 200 °C, the lowest concentrations of linoleic acid were obtained after 1 min, 15 min and 10 min with 0.87, 0.37, and 0.25 mg/mL, respectively. We presumed that polyunsaturated fatty acids may be converted to linoleic acid at the initial stage of the reaction, and then linoleic acid continues to oxidize to form saturated fatty acids at sustained high temperatures. Because the concentration of linoleic acid was unstable, and the temperature dependence of the reaction was complicated, the Arrhenius equation was not suitable for this case.

### Kinetic Changes of α-Dicarbonyl Compounds Concentrations in Model System

**Figure 2:**
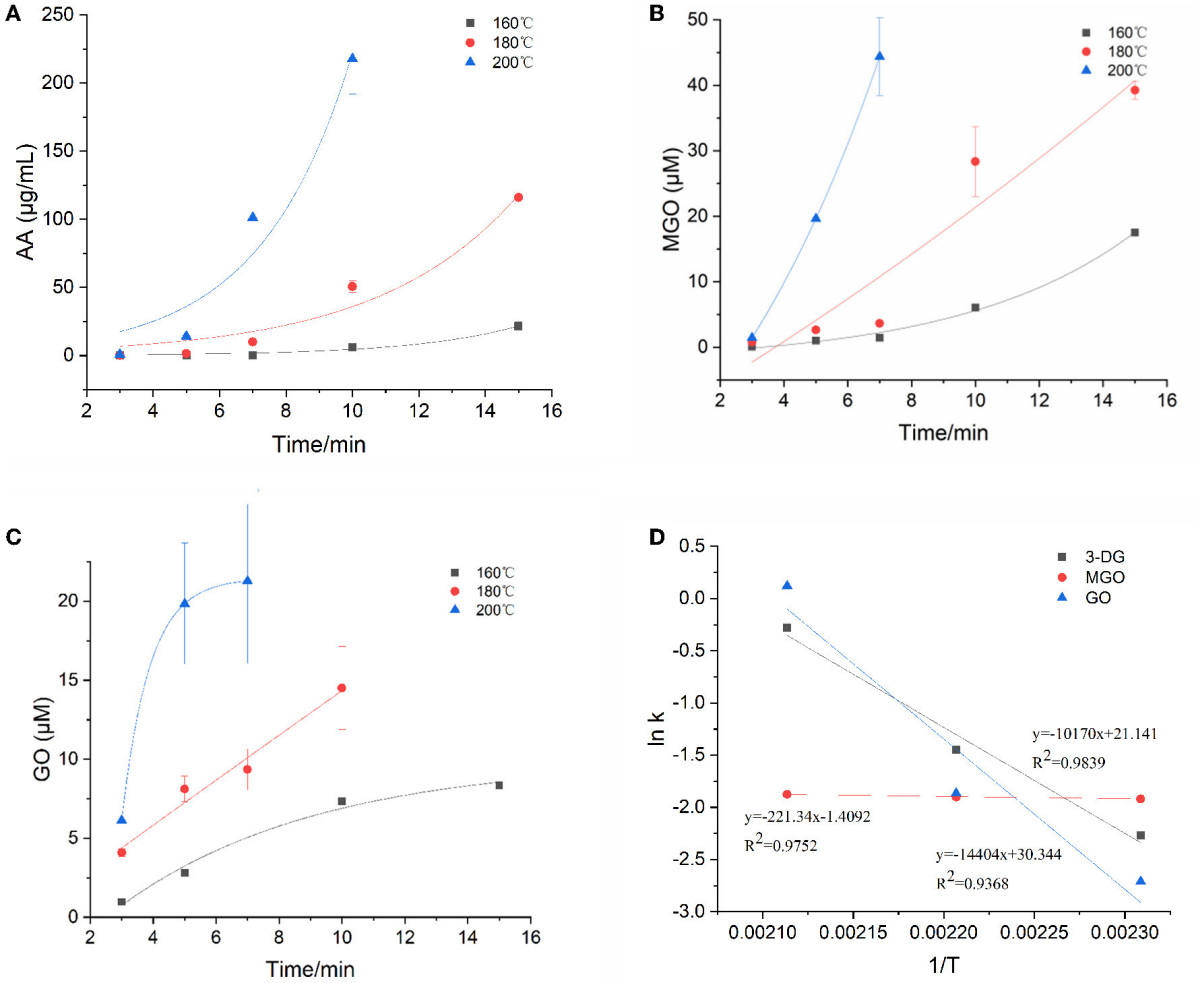
Kinetic changes of α-dicarbonyl compounds in heated model system **(A)** 3-DG, **(B)** MGO, **(C)** GO, and **(D)** Arrhenius plot.

α-Dicarbonyl compounds such as 3-DG, MGO, GO are key reaction intermediates formed during caramelization and Maillard reactions in food processing. The concentrations of three main α-dicarbonyl compounds (3-DG, MGO and GO) in the model system at three temperatures were detected and then the kinetic fit plots were drawn (Figures 2A–C). They reached their maximum concentration at a corresponding time points under three heating temperatures, and the maximum concentration increased with increasing temperature. In general, their maximum concentration was in the order as MGO > 3-DG > GO (except for MGO at 160°C). Similar results were previously reported for theα-dicarbonyl compounds formation of coffee during roasting at 220 and 240 °C, which was indicated that formation of MGO predominantly through 3-DG (16).

The degradation of Amadori products and Heyns products leads to the formation of 3-DG (25). In addition, 3-DG can also be formed by removing one molecule of water from glucose during caramelization (3). As shown in Figure 2A, at the three heating temperatures, 3-DG increased instantly with the extension of heating time, and appeared to be flat at the later stage of reaction at 200°C, and then gradually decreased. 3-DG reached the highest concentration when heated at 160°C for 15 min, 180 °C for 10 min and 200°C for 7 min, which were 24.7 μM, 25.5 μM and 26.4 μM, respectively. The formation of MGO could be a result of the cleavage of Schiff base into C2 and C3 compounds (26). In addition, it has been reported that MGO formation occurs due to the cleavage of the C3-C4 bond in 1-deoxyglucosone (1-DG) (3). Besides, Thornalley et al. (25) also reported that MGO can be formed from 3-DG by retroaldol cleavage. As shown in Figure 2B, with the increase of heating temperature and time, the increasing rate of MGO was gradually accelerated, which was faster than that of 3-DG. In the early stage of the reaction at the three temperatures, the concentrations of 3-DG (0.3, 1.9, 7.1 μM) were higher than that of MGO (0.1, 0.8, 1.4 μM). But as the reaction progressed, the maximum concentrations of MGO were about 1.5 times higher than 3-DG at the late reaction stage. GO was one of the α-dicarbonyl compounds observed in the samples, but its content was lower than that of 3-DG and MGO. It is formed due to the degradation of Amadori products or deoxyglycosides (27). In addition, it can also be directly formed from glucose or imine via retroacetalization followed by oxidation (25). As shown in Figure 2C, only at high temperature of 200°C the reaction rate of GO increased sharply at the beginning of the reaction and then leveled off. Its concentration at later stages of the reaction was lower than that of 3-DG and MGO.

The kinetic changes of α-dicarbonyl compounds during the heating process could be expressed by the first-order reaction kinetic model, which were all in line with the Exponential equation. The model parameters fitted according to the measured concentrations of the reaction were shown in Table 2. It is observed that R^2^ > 0.915 for the fitted curves of 3-DG, R^2^ > 0.819 for MGO and R^2^ > 0.922 for GO, indicating that the models can describe the concentration changes of the reaction well. The kinetic rate constants k of each substance was gradually increasing with the temperatures, which indicated that high temperature promoted the reaction. The Arrhenius plots of 3-DG, MGO and GO were shown in Figure 2D. According to the slope of the linear equation, the activation energy E_a_ of the reaction can be calculated, and E_a_ of 3-DG, MGO and GO were 84.55, 1.84, and 119.75 kJ/mol, respectively. According to the E_a_ values, MGO is more sensitive to temperature than 3-DG and GO.

**Table 2:** Parameter of kinetics of α-dicarbonyl compounds in heated model system.

	**Temperature (**°**C)**	**k**	**R^**2**^**	**E_**a**_ (kJ/mol)**
3-DG	160	0.103	0.915	84.55
	180	0.235	0.917	
	200	0.758	0.917	
MGO	160	0.147	0.991	1.84
	180	0.149	0.819	
	200	0.153	0.819	
GO	160	0.067	0.961	119.75
	180	0.155	0.922	
	200	1.128	0.922	

### Kinetic Changes of AA and 5-HMF Concentrations in Model System

**Figure 3:**
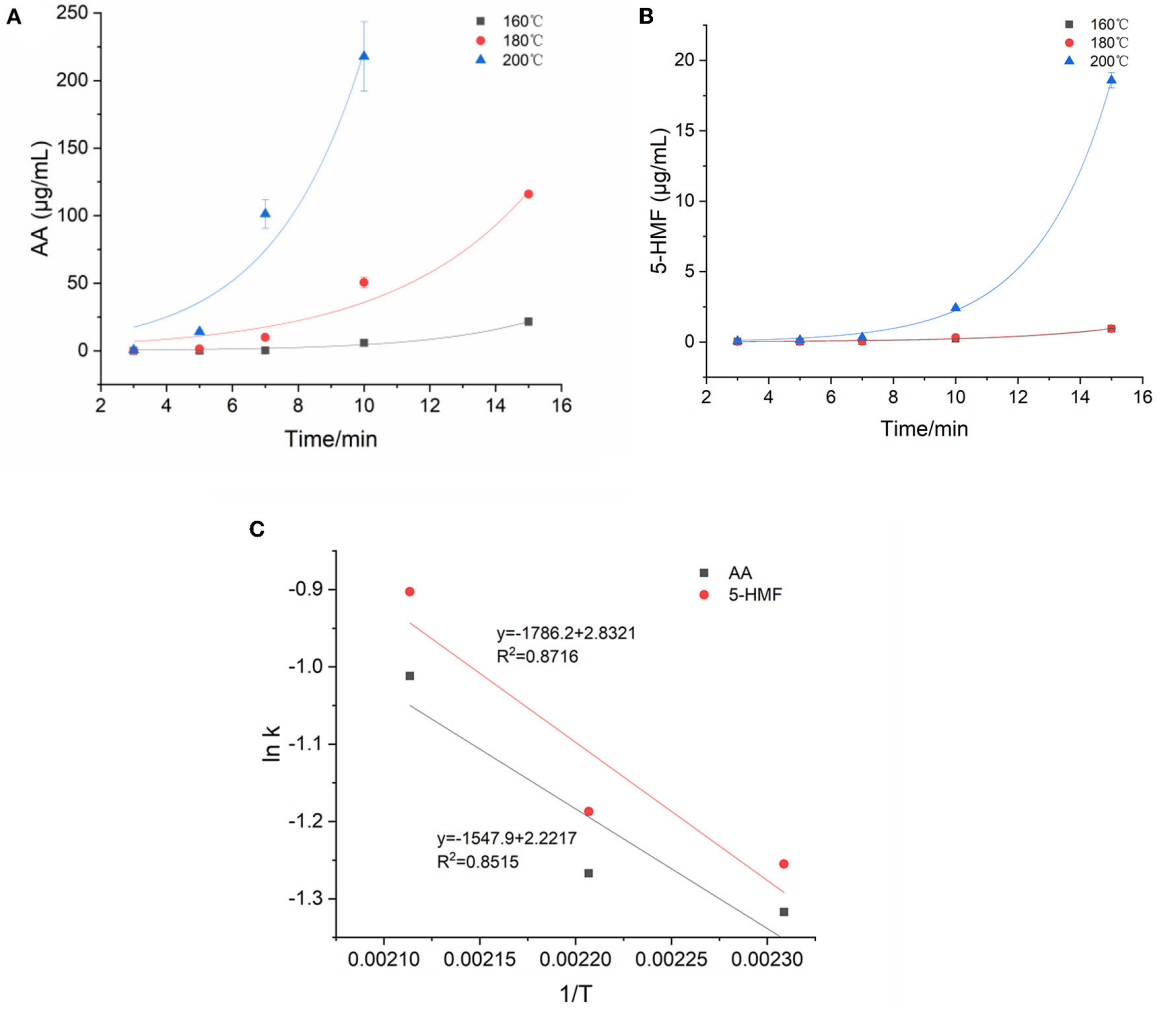
Kinetic changes of AA and 5-HMF in heated model system **(A)** AA, **(B)** 5-HMF, and **(C)** Arrhenius plot.

Figure 3A showed the kinetic fitting diagram of AA. Throughout the reaction process, AA showed an upward trend with the heating time, and at 160°C, the AA concentration gradually increased after a longer lag period of 10–15 min, but increased sharply at 180 and 200°C, indicating that the concentration of AA was greatly affected by time and temperature. The concentration increased by >276–2,000 times from the start of the reaction to the highest point of the reaction. Parker et al. (28) called the direct reaction of asparagine with reducing sugar to generate AA as the specific amino acid pathway. In addition to specific amino acid pathways, α-dicarbonyl or hydroxycarbonyl compounds formed from the dehydration of Amadori products of reducing sugars and amino acids may also react with asparagine to form AA. They called these AA formation pathways the universal amino acid pathway. By monitoring the concentration changes of 5-HMF during the reaction process, the reaction kinetic diagram was fitted based on the measured value of the concentration (Figure 3B). Although the dynamic change of 5-HMF was similar to that of AA, the concentration increased to the maximum value with the increasing temperature and time, the temperature and heating time had no significant effects on the concentration of 5-HMF when heated below 200°C. Moreover, at 200°C, 5-HMF increased sharply in the late reaction phase, and the maximum concentration was nearly 20 times higher than that at 160 and 180°C, which suggested that 5-HMF was easier to form at temperature higher than 200°C. In this model, the concentration of AA is 10–100 times higher than that of 5-HMF. Gokmen et al. (29) reported that 5-HMF with a carbonyl function was able to form AA in a low-moisture model system by reacting with asparagine. This may explain the above difference of concentration between AA and 5-HMF.

The kinetic changes of AA and 5-HMF during the heating process were both described by the first-order reaction kinetic model (Exp2PMod1). The kinetic parameters were shown in Table 3. Unsurprisingly, the kinetic rate constants (k) of the two target hazards increased with increasing temperature, with k values ranging from 0.268 to 0.364 and 0.285-0.405min^−1^ for AA and 5-HMF. Obviously, the reaction rate of 5-HMF was higher than that of AA. It was observed from Table 3 that the concentration changes of AA and 5-HMF fitted the curve with R^2^ > 0.926, indicating that the model could accurately describe the concentration changes of the reaction, confirming that their generation followed a first-order reaction. The temperature dependence of AA and 5-HMF was estimated using the Arrhenius equation and shown in Figure 3C. The activation energy E_a_ of the reaction was calculated according to the slope of the linear equation, and the E_a_ of AA and 5-HMF were calculated to be 12.87 and 14.85 kJ/mol, respectively, which indicated that the formation reaction of hazardous substances was more likely to occur at high temperature. In contrast to the previous overview of AA formation in various model systems and corresponding E_a_ (48.5–277 kJ/mol) by De Vleeschouwer et al. (30), much lower E_a_ was obtained for our study, which probably resulted from the lower concertration of reactants we used. Meanwhile, a study about coffee showed that the E_a_ values for the formation 5-HMF was calculated to be 47.31 117.38 kJ mol^−1^ (31), which can be explained by the matrix effect of the coffee system.

**Table 3:** Parameter of kinetics of AA and 5-HMF in heated model system.

	**Temperature (**°**C)**	**k**	**R^**2**^**	**E_**a**_ (kJ/mol)**
AA	160	0.268	0.980	12.87
	180	0.282	0.944	
	200	0.364	0.926	
5-HMF	160	0.285	0.996	14.85
	180	0.305	0.974	
	200	0.405	0.999	

### Correlation Analysis Between Precursors, α-Dicarbonyl Compound and Target Hazards

**Figure 4:**
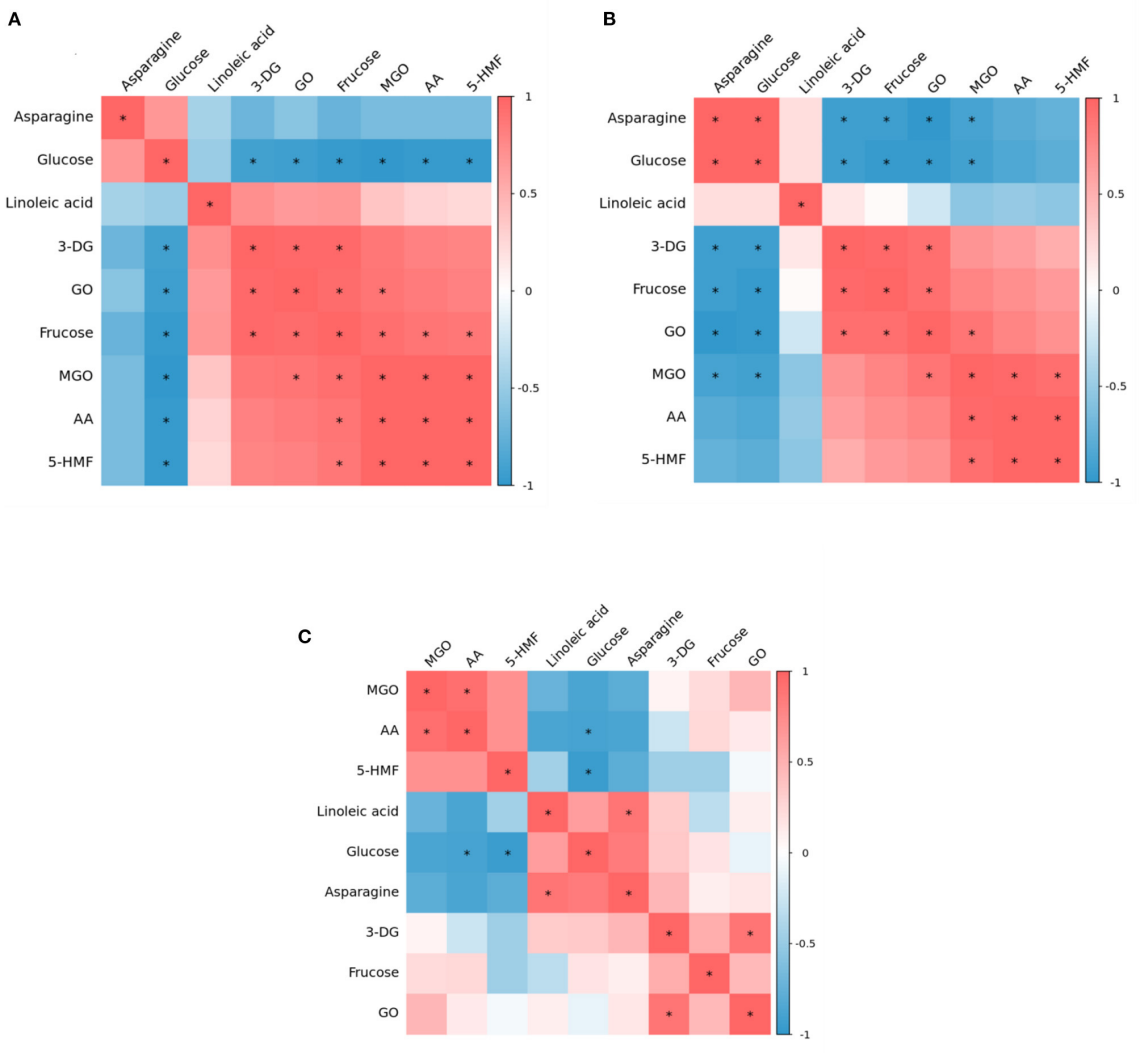
Correlation heat map among precursor, intermediates and contaminants in heated model system **(A)** 160 °C, **(B)** 180 °C, and **(C)** 200 °C.

Maillard, caramelization and lipid oxidation almost simultaneously exist in food systems, thus they may interact with each other. Therefore, heat maps of correlation analysis (Figure 4) were constructed based on the concentrations of precursors (glucose, fructose, asparagine, linoleic acid), α-dicarbonyl compounds (3-DG, GO, MGO) and hazardous substances (AA and 5-HMF) at three heating temperature to identify possible correlations between various parameters in the glucose-asparagine-linoleic acid model system. This analysis was carried out using a Pearson's correlation test for the significance assessment.

At 160°C, glucose and asparagine were positively correlated with each other, but negatively correlated with other substances, among which glucose had a significant negative correlation with all detected intermediates and hazardous substances, which proved that in the model system, glucose should be the main precursor for the formation of intermediates and hazardous substances. Nguyen et al. have found glucose can produce the highest AA and 5-HMF concentration compared to other sugars (6). Interestingly, linoleic acid and fructose were observed to have positive correlations with each intermediate and two hazards. Meanwhile, there were significant positive correlations between 3-DG and GO, GO and MGO, MGO and AA, MGO and 5-HMF, and AA and 5-HMF. It was reported that both of glucose and fructose can generate 5-HMF through the formation of a dicarbonyl intermediate, 3-DG, from the Maillard reaction and caramelization. Thus, we presumed that the formation of AA and 5-HMF from a generic amino acid route with glucose and fructose simultaneously involved (but glucose as the main precursor) played a crucial role at this stage of lower temperature.

Whereas at 180°C, it is clear from Figure 4B that the correlation between the various substances was weakened. Besides, at 180°C glucose and asparagine became significantly positively correlated with each other, and the negative correlation between asparagine and 3-DG, fructose, GO, MGO became significant. However, glucose still negatively correlated with AA and 5-HMF, but not significantly. The correlations between 3-DG, fructose, GO, MGO, AA and 5-HMF were the same as at 160°C, but the positive correlations of fructose with MGO, AA and 5-HMF became insignificant. It was reported that in the specific amino acid route, reducing sugars react with asparagine to form the Schiff base that is thereafter decarboxylated to form acrylamide, without rearrangement of the Amadori products and fragmentation of sugar (28). Thus it was possible that at this stage the specific amino acid route may play a dominant role in the AA and 5-HMF formation.

As shown in Figure 4C, at 200°C, the correlations between various parameters gradually weakened, among which, the correlations of 3-DG, fructose and GO concentration with other substances were not significant. It is worth mentioning that at 180 and 200°C, the relationship of linoleic acid with AA and 5-HMF changed from a positive correlation at 160°C to a negative correlation, and the negative correlation with AA was stronger in comparison, indicating that under such high temperature conditions, in addition to the glucose and asparagine pathways, the linoleic acid pathway may be crucially involved for the formation of AA and 5-HMF.

## Conclusion

This study systematically studies kinetic changes of the precursors (glucose, fructose, asparagine, linoleic acid), α-dicarbonyl compound intermediates (3-DG, MGO, GO) and target hazards (AA and 5-HMF) in the “glucose-asparagine-linoleic acid” ternary system. The kinetics analysis suggested that MGO was more likely to be formed during the reaction. The formation of AA and 5-HMF both obeyed first-order kinetics. In addition, the kinetic reaction rate constants under different heating temperatures suggested the formation of AA and 5-HMF were temperature dependent. Furthermore, the correlation analysis between precursors, intermediates and target hazardous substances indicated that at lower temperature, the generic amino acid route and the specific amino acid route may paly crucial roles for the formation of AA and 5-HMF. With the temperature increased, the relationship between linoleic acid and AA and 5-HMF changed from a positive correlation at low temperature to a negative correlation, indicating that at this high temperature, except for glucose and asparagine pathways, there may also be a linoleic acid pathway for the generation of AA and 5-HMF.

## Data Availability Statement

The original contributions presented in the study are included in the article/supplementary material, further inquiries can be directed to the corresponding authors.

## Author Contributions

YM: methodology, investigation, data curation, software, and writing—original draft. YL: software and investigation. YZ: investigation. FL: data curation. BG: validation. QY: project administration, validation, and formal analysis. JX: resources and supervision. YC: conceptualization, writing—review and editing, visualization, project administration, and funding acquisition. All authors contributed to the article and approved the submitted version.

## Funding

The financial supports from the National Key Research and Development Program of China (2019YFE0106000 and 2017YFC1600405), the Technology Innovation Leading Program of Jiangxi (20212BDH80001), and the Open Project Program of State Key Laboratory of Food Science and Technology, Nanchang University (No. SKLF-KF-202011) are gratefully acknowledged.

## Conflict of Interest

The authors declare that the research was conducted in the absence of any commercial or financial relationships that could be construed as a potential conflict of interest.

## Publisher's Note

All claims expressed in this article are solely those of the authors and do not necessarily represent those of their affiliated organizations, or those of the publisher, the editors and the reviewers. Any product that may be evaluated in this article, or claim that may be made by its manufacturer, is not guaranteed or endorsed by the publisher.
